# Assessment of *Helicobacter pylori* Prevalence in Fresh Raw Meat: Species and Source-Based Analysis

**DOI:** 10.3390/microorganisms14020379

**Published:** 2026-02-05

**Authors:** Dhary A. Almashhadany, Sara M. Mayas, Abdulwahed A. Hassan, Izhar U. H. Khan

**Affiliations:** 1 Department of Medical Laboratory Science, College of Science, Knowledge University, Erbil 44001, Iraq; dhary.alewy@knu.edu.iq; 2 Department of Microbiology, Faculty of Applied Science, Thamar University, Dhamar 00967, Yemen; saramayas@tu.edu.ye; 3 Medical Laboratories Department, Alnoor University, Mosul 41002, Iraq; 4 Department of Veterinary Public Health (DVPH), College of Veterinary Medicine, University of Mosul, Mosul 41003, Iraq; 5 Agriculture and Agri-Food Canada, Ottawa Research and Development Centre, 960 Carling Ave., Ottawa, ON K1A 0C6, Canada

**Keywords:** *Helicobacter pylori*, raw meat, foodborne pathogens, prevalence, Yemen

## Abstract

*Helicobacter pylori* is a major causative agent of chronic gastritis, gastric and duodenal ulcers, gastric cancer, and mucosa-associated lymphoid tissue lymphoma. Transmission to humans may occur through the consumption of contaminated food. This study investigated the prevalence of *H. pylori* in fresh raw meat from different animal sources and assessed the efficiency of selective media for its recovery in Dhamar Governorate, Yemen. A total of 380 meat samples, including beef (*n* = 125), sheep (*n* = 135), and goat (*n* = 120), were collected from slaughterhouses (*n* = 127), retail markets (*n* = 124), and butcher shops (*n* = 129). Three selective media: Modified Campy-blood Agar (MCA), Belo Horizonte Agar (BHA), and Egg Yolk Emulsion (EYE) medium were evaluated for comparative recovery from each meat source. Overall, *H. pylori* was detected in 47 samples (12.4%), with a relatively high prevalence in beef (15.2%), followed by goat (12.5%) and sheep (9.6%). By source, butcher shop samples comparatively showed the highest prevalence (15.5%), especially in beef (19.1%), goat (14.3%), and sheep (13.3%), as compared to the slaughterhouses (11.8%) and retail markets (9.7%). Differences among meat types and sources were not statistically significant (*p* = 0.394 and *p* = 0.362). Overall, selective media comparison revealed that MCA showed a relatively high recovery rate (6.6%), followed by EYE (3.4%) and BHA (2.4%). A seasonal trend was observed, with peak prevalence in April (20%). These findings suggest that raw meat may represent a potential source of exposure to *H. pylori*, although its role in transmission to humans remains to be fully clarified. Regular monitoring, improved hygiene practices, and stricter control of environmental contamination are recommended to reduce associated public health risks.

## 1. Introduction

*Helicobacter pylori* is a fastidious Gram-negative, microaerophilic, rod-shaped, spiral motile bacterium that possesses four to six unipolar-sheathed flagella. This may be an adaptation to survive in gastric juices [1]. Under stress conditions such as insufficient nutrients, desiccation, exposure to increased levels of oxygen, and exposure to antimicrobial agents, *H. pylori* can enter a viable but non-culturable (VBNC) state and change its rod-like shape to a coccoid shape with high motility, where a spiral or curved morphology serves as evidence [2]. *H. pylori* colonies can be phenotypically identified by confirming the presence of urease, catalase, and oxidase enzymes [3]. Culture-based recovery of *H. pylori* from food matrices likely underestimates its true prevalence due to fastidious growth requirements, competition with abundant background microflora, and its ability to enter VBNC state under environmental stress. Therefore, confirmation of *H. pylori* detection and identification to species-level is typically based on 16S rRNA gene, whereas other genes such as *ureA, glmM,* or *cagA* can be used to assess virulence factors [4].

*H. pylori* is one of the most common human pathogens, with a reported 4.4 billion global infections [5]. *H. pylori* is the causative agent of upper gastrointestinal disorders, including chronic gastritis, peptic ulcers, gastric mucosa-associated lymphoid tissue (MALT), and gastric carcinoma. It is classified as a Group I carcinogen that can have a carcinogenic effect on humans [6]. *H. pylori* can form biofilms on gastric mucosa and abiotic surfaces, enhancing its persistence and pathogenicity [7]. Moreover, biofilms protect bacteria from gastric acidity, oxidative stress, host immunity, and antibiotics, often leading to treatment failure and infection recurrence [8]. Approximately 15–20% of individuals infected with *H. pylori* develop peptic ulcers, whereas the majority of infections remain asymptomatic [9]. The prevalence of *H. pylori* infection varies depending on the geographical region and study period. Generally, a higher incidence of *H. pylori* is reported in developing as compared to industrialized countries. These differences can be correlated with socio-economic status, family income levels, hygiene, and housing conditions of the population [10].

The routes of *H. pylori* transmission from human to human have not been fully determined, but the bacterium is usually transmitted through direct routes, including oral–oral, fecal–oral, gastro–oral and sexual routes; however, anal–oral and genital–oral pathways have not been considered as a principal route of transmission [11,12]. The bacterium can also be transmitted to humans through indirect routes, such as consumption of contaminated food or water; however, this transmission route has not been extensively studied. A study has indicated the possible transmission of *H. pylori* via drinking water [13]. *H. pylori* has been detected in cow, sheep, goat, buffalo and camel meat samples and minced beef [2], and various types of shellfish, dry fermented sausages, milk and dairy products, vegetables, fruit, and fruit juices [14,15,16]. Moreover, *H. pylori* has been recovered from tap, well water, and various environmental water sources. This indicates that it can survive in water for long periods, and can be an important reservoir and route for *H. pylori* infection [13,17,18,19]. Moreover, other study findings also support the hypothesis of *H. pylori* as a zoonotic pathogen, as it has been detected in different farm and domestic animals [20]. In a recent study, *H. pylori* was isolated from both human and animal samples, suggesting that pet and farm animals could potentially be a source of transmitting *H. pylori* infection to humans [21].

Yemen has limited surveillance data on *H. pylori*, particularly in relation to its presence in food sources such as raw meat. Traditional meat handling practices, limited regulatory oversight, and gaps in public health infrastructure further elevate the risk of foodborne transmission. This study addresses a critical knowledge gap and contributes to a better understanding of foodborne pathogen food safety in underrepresented regions.

Therefore, the objectives of the present study were to: (a). Investigate and determine the prevalence rate of *H. pylori* in fresh raw meat of slaughtered large (beef cattle) and small (sheep and goat) livestock animals collected from slaughterhouses, retail markets, and butcher shops; and (b). Assess and compare the performance of three selective growth media for enhanced recovery and isolation of *H. pylori*. The findings will aid in understanding potential contamination pathways, evaluating *H. pylori*’s persistence in food matrices, recovery efficiency on selective media, and characterizing antimicrobial resistance patterns as part of One Health surveillance. This perspective aligns with the role of veterinary public health in preventing zoonotic transmission, safeguarding food supply chains, and informing risk-based hygiene interventions.

## 2. Materials and Methods

### 2.1. Sample Collection

A total of 380 fresh raw meat samples, including beef (*n* = 125), sheep (*n* = 135), and goat (*n* = 120), were collected monthly between January and June 2021 from slaughterhouses (*n* = 127), retail markets (*n* = 124), and butcher shops (*n* = 129) located in Dhamar Governorate, Yemen (Table 1). From each carcass, approx. 50 g was excised from five different anatomical sites, including shoulder, breast/foreshank, rack, loin and leg. These portions were combined to obtain a composite sample of 250 g per carcass. All samples were aseptically placed into sterile polyethylene bag, transported in a cooler, and delivered to the Department of Microbiology, Faculty of Applied Sciences, Thamar University. The samples were processed and analyzed within 24 h of their collection.

### 2.2. Culture Conditions for Isolation and Recovery of H. pylori

For the isolation and recovery of *H. pylori*, 50 g of each meat sample was finely chopped using sterile blades and homogenized in 250 mL of 0.9% NaCl solution. All procedures were performed under strict aseptic conditions to prevent cross-contamination. The suspension was gently mixed and left to stand at room temperature for 10 min. Since *H. pylori* is a fastidious and slow-growing bacterium, a prolonged enrichment step was employed to inhibit other background microflora and enhance the recovery of stressed and/or viable-but-non-culturable (VBNC) cells of *H. pylori*. A 2 mL aliquot of the homogenate was transferred into 18 mL of Brain Heart Infusion (BHI) broth (Oxoid, Wesel, Germany) supplemented with 7% horse serum and incubated at 37 °C for 7–10 days under microaerophilic (85% N_2_, 10% CO_2_, 5% O_2_) condition using the CampyGen GasPak system (Oxoid, Basingstoke, UK). Following enrichment, 100 µL suspension was plated in duplicate on three selective media: Modified Campy-blood Agar (MCA) (HIMEDI, Mumbai, India) containing 10% sheep blood; Belo Horizonte Agar (BHA) (HIMEDI) composed of a brain–heart infusion base (0.4%), 3,4,5-triphenyltetrazolium chloride (0.4%) (HIMEDI), and 10% sheep blood; and Egg Yolk Emulsion (EYE) medium (Oxoid), comprising Columbia agar (HIMEDI) with 10% egg yolk emulsion, 1% Vitox (Oxoid), and 2,3,5-triphenyltetrazolium chloride (40 mg/L) (HIMEDI). All plates were incubated at 37 °C for 7–10 days under microaerophilic conditions [22,23,24]. Plates were examined periodically after 72 h of incubation, and putative *H. pylori* colonies were subcultured to fresh media to prevent overgrowth by competing microorganisms and to maintain culture purity. To ensure comparability of recovery efficiency, each sample was inoculated on all three selective media, with the same number of samples tested per medium.

### 2.3. Phenotypic Identification of Isolates

The isolates obtained from the same sample, regardless of the selective media used, were considered to be of common origin and used as a single strain. Putative *H. pylori* colonies were initially identified based on colony morphology (size, color, texture, and growth pattern) and then sub-cultured on MCA for purification. Isolates were incubated at 37 °C for 48–72 h under microaerophilic conditions. For initial confirmation, colonies were subjected to a modified Gram staining reaction as described by Logan and Walker [24]. Briefly, the cells were fixed with methanol instead of heat, decolourisation for 2–5 s. instead of 15–30 s. and staining with safranin for 3 min. The standard biochemical tests, including catalase, oxidase, urease, and indole production, were performed. Selective growth tests were performed using media supplemented with 1% glycine and 3.5% NaCl. In addition, antibiotic susceptibility patterns were used to distinguish *H. pylori* from closely related species. *H. pylori* is typically resistant to nalidixic acid and susceptible to cephalothin, in contrast to many *Campylobacter* species, which show the opposite pattern [25].

### 2.4. Antibiotic Susceptibility Testing

To avoid overestimation of prevalence and potential bias in antimicrobial susceptibility testing, only one representative isolate per sample was included in the analysis. In addition to the nalidixic acid and cephalothin susceptibility testing, the following antimicrobial agents, according to the international guidelines, were included in this analysis: ampicillin (AMP, 10 µg), amoxicillin (AMX, 10 µg), erythromycin (E, 15 µg), tetracycline (TE, 30 µg), metronidazole (MT, 5 µg), clarithromycin (CLR, 15 µg), levofloxacin (LE, 5 µg), and azithromycin (AZM, 30 µg). Since disk diffusion has limitations for *H. pylori*, as CLSI (Clinical and Laboratory Standards Institute) (2023) M100 [26] and the EUCAST (European Committee on Antimicrobial Susceptibility Testing) (2023) [27] do not currently provide standardized zone-diameter breakpoints for *H. pylori*. Consequently, interpretations based on disk diffusion may lack accuracy and reproducibility, and results should be considered indicative rather than definitive. Therefore, antibiotic susceptibility was assessed using the modified Kirby–Bauer disk diffusion method on Mueller–Hinton agar (Oxoid, UK). Zones of inhibition were measured after incubation under microaerophilic conditions, and interpretation of susceptibility was based on cut-offs described by Chaves et al. [28]. Quality control was ensured by including reference strains, verifying disk and media performance, and performing replicate assays.

### 2.5. Nucleic Acid Extraction and Species-Specific PCR Assay

Each isolate was further confirmed as *H. pylori* by PCR assay. For DNA extraction, a few pure colonies from each isolate were suspended in 180 µL of 1× TE buffer, boiled at 100 °C for 10 min, and then centrifuged at high speed. The resulting supernatant was used as the DNA template for PCR amplification. The extracted DNA was further confirmed using a *H. pylori* species-specific polymerase chain reaction (PCR) targeting a conserved region of the 16S rRNA gene, as described by Clayton et al. [29]. Prior to the PCR assay, the specificity of the primers was further validated through the BLAST search against the available NCBI nucleotide database. The PCR assay was conducted after confirming 100% sequence identity of the primers with *H. pylori* and observing no significant alignment with other *Helicobacter* or phylogenetically related bacterial species. A 25 µL PCR mixture contained 20–40 ng of template DNA, 1.25 U of Ex Taq DNA polymerase, 1× PCR buffer, 25 mM MgCl_2_, and 10 µM of each forward: Hp1 (5′-CTG GAG AGA CTA AGC CCT CC-3′) and reverse: Hp2 (5′-ATT ACT GAC GCT GAT TGT GC-3′) primers. The PCR was performed using an initial denaturation at 94 °C for 3 min, followed by 35 cycles consisting of denaturation (94 °C for 30 s), annealing (55 °C for 30 s), and extension (72 °C for 45 s) steps ending with a 5 min final extension at 72 °C [29]. The PCR amplicons, with an expected 520 bp fragment specific to *H. pylori*, were electrophoresed on a 1.5% agarose gel matrix with 1× TAE buffer using a 1 kb DNA size marker. The gel was stained in 0.5 μg mL^−1^ ethidium bromide solution. The gel was visualized on an ultraviolet (UV) transilluminator and photographed.

### 2.6. Statistical Analysis

Prevalence rates and comparisons across meat types, sources, and selective media were evaluated using Pearson’s Chi-square and Fisher’s Exact Tests, with a significance threshold set at *p* < 0.05. Confidence intervals (CIs) at 95% confidence level were calculated to estimate variability in the sample mean and standard deviation.

## 3. Results

### 3.1. Growth Pattern and Recovery on Selective Media

Based on colony morphology, Gram staining, and biochemical profiles, *H. pylori* isolates were identified. *H. pylori* formed small, round, translucent colonies on MCA, as compared to BHA (supplemented with tetrazolium chloride), where colonies appeared golden due to dye reduction. On the other hand, red colonies developed against a yellow background on EYE medium, also resulting from tetrazolium dye reduction. Overall, of the total 380 meat samples, 64 putative culture isolates (beef: 24; sheep: 19; and goat: 21) were recovered from MCA, EYE and BHA selective growth media where MCA showed a relatively high recovery rate (*n* = 25; 6.6%), followed by EYE (*n* = 13; 3.4%) and BHA (*n* = 9; 2.4%). Of these 64 culture isolates, 47 (73.4%) isolates were confirmed as *H. pylori* through biochemical characterization, including Gram staining showed the typical curved rod morphology, and all isolates were positive for catalase, oxidase, urease, and H_2_S production in TSI with lead acetate; negative for indole; and positive for hippurate hydrolysis. Growth was observed in media containing 1% glycine and 3.5% NaCl. Species identity was further confirmed by *H. pylori*-specific PCR targeting the partial 16S rRNA gene, where all isolates produced the expected ~520 bp amplicons. Of the total 47, 19 isolates from beef, 13 from sheep, and 15 from goat meat samples were positive (Table 1). The 47 confirmed isolates were recovered from different media, 25 (53.2%) from MCA, 13 (27.7%) from EYE, and 9 (19.1%) from BHA. The results showed that the recovery rates differed significantly (χ^2^ = 8.85, df = 2, *p* = 0.012) among the three media. Specifically, MCA exhibited a significantly higher recovery efficiency compared to BHA and EYE, confirming its superior performance in isolating *H. pylori* in this study.

### 3.2. Detection and Prevalence of H. pylori

**Figure 1:**
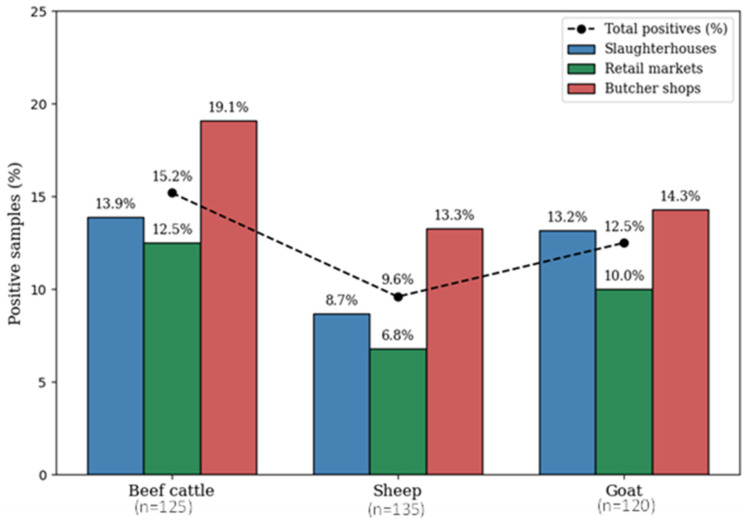
Prevalence of *H. pylori* in beef, sheep, and goat meat samples in various sources (slaughterhouse, retail market, and butcher shop). The dashed line indicates the total number of positive samples per meat type. Bars represent prevalence (%) of confirmed isolates within each group. Percentages were calculated relative to the total samples tested in each group and are shown above each bar.

Among the 380 raw meat samples, 47 tested positive for *H. pylori*, yielding an overall 12.4% prevalence. Prevalence differed among meat types, with high prevalence in beef (15.2%; *n* =19), followed by goat (12.5%; *n* = 15) and sheep (9.6%; *n* = 13) (Table 1). Statistical analysis showed no significant (*p*-value = 0.394) difference among meat types. When comparing sources of meat, prevalence was relatively higher in butcher shops (15.5%; *n* = 20) as compared to slaughterhouses (11.8%; *n* = 15) and retail market (9.7%; *n* = 12) (Table 1; Figure 1). However, these differences were not statistically significant (*p*-value = 0.362), suggesting similar contamination levels across sources.

### 3.3. Antimicrobial Susceptibility

All *H. pylori* isolates were resistant to nalidixic acid and susceptible to cephalothin. Among the other antibiotics tested, the highest susceptibility rates were observed for amoxicillin (AMX, 80.8%), levofloxacin (LE, 78.7%), ampicillin (AMP, 76.6%), and clarithromycin (CLR, 74.5%). Moderate susceptibility was noted for azithromycin and tetracycline (AZM, TE 61.7% each), while metronidazole and erythromycin showed comparatively lower susceptibility (MT, 53.2% and E, 48.9%, respectively). Resistance-level ranged from 12.8% for amoxicillin to 40.4% for metronidazole, with intermediate resistance generally low (4.3–14.9%). These results indicate a variable resistance profile across different antibiotic classes, highlighting both therapeutic options and emerging resistance trends (Table 2).

By sample origin, beef isolates displayed the highest resistance, particularly to erythromycin and metronidazole, while sheep isolates showed the lowest resistance among most antibiotics. Goat isolates were characterized by elevated resistance to levofloxacin and tetracycline. These host-related differences suggest possible variation in antibiotic exposure or selective pressure among animal sources (Table 2). However, there was no statistical significance between meat source and antimicrobial resistance profile observed.

### 3.4. Descriptive Statistics

As shown in Table 1, the prevalence of *H. pylori* varied slightly among meat types and sampling sources. Although beef samples exhibited the highest prevalence and sheep the lowest, the overlapping confidence intervals and results of statistical analysis indicated that these differences were not statistically significant (*p* > 0.05). On the other hand, butcher shops exhibited the highest proportion of positive samples, followed by slaughterhouses and retail market. However, analysis revealed no significant differences in prevalence among the different sources (*p* > 0.05). This indicates that *H. pylori* contamination is relatively consistent across different meat sources and retail settings (Table 1).

### 3.5. Temporal Distribution of H. pylori Prevalence

The temporal distribution of *H. pylori*-positive samples is summarized in Table 3. The highest occurrence was recorded in April (20%; *n* = 13), followed by May (16.9%; *n* = 11) and June (16.1%; *n* = 10), whereas the lowest prevalence was observed in February and March (6.3% each; *n* = 4). Although prevalence appeared to increase during late spring, statistical analysis indicated that these monthly differences were not significant.

Regarding the source of meat samples, butcher shops exhibited the highest contamination rate with *H. pylori* (15.5%; *n* = 20), followed by slaughterhouses (11.8%; *n* = 15) and retail markets (9.7%; *n* = 12). Similarly, when analyzed by meat type, beef samples showed the highest prevalence (15.2%; *n* = 19), compared to goat (12.5%; *n* = 15) and sheep (9.6%; *n* = 13). Despite these observed differences, overall, statistical analysis indicated that variations in *H. pylori* prevalence among meat types, sources and months were not statistically significant (*p* > 0.05) (Table 3).

## 4. Discussion

Livestock and poultry naturally harbor intestinal microorganisms such as *Campylobacter* spp., *Escherichia coli*, and *Salmonella*, which can spread to carcasses during slaughtering and evisceration [23,30]. In contrast, *H. pylori* is a fastidious microaerophilic bacterium requiring specific conditions (5–10% O_2_, 10% CO_2_, and 37 °C) that are unlikely to persist in slaughterhouse environments [31]. Nevertheless, its detection in raw meat samples suggests possible fecal or environmental contamination during processing, underscoring the importance of strict hygiene measures at both farm and slaughterhouse levels [32,33].

In the present study, *H. pylori* recovery was attempted using three selective media MCA, BHA, and EYE. Although MCA yielded the highest proportion of isolates (53.2%), this difference was statistically significant (*p* = 0.012), indicating superior recovery efficiency as compared to EYE and BHA. Earlier, Piccolomini et al. [34] reported higher recovery of *H. pylori* from gastric biopsies using EYE and modified chocolate agar as compared to other selective and nonselective media, highlighting the influence of specimen type and medium composition. Importantly, Stevenson et al. [35] developed a selective *H. pylori* Special Peptone agar (HPSPA) specifically selective to isolate *H. pylori* from potential animal and food sources, demonstrating that highly selective formulations containing multiple antimicrobial agents can enhance recovery while minimizing background flora. Moreover, media supplemented with 1% glycine and 3.5% NaCl are designed to suppress competing background flora while promoting the selective recovery of *H. pylori* without compromising its viability. Glycine disrupts bacterial cell wall synthesis, effectively inhibiting many non-target microorganisms. However, *H. pylori* remains unaffected due to its unique adaptive mechanisms, including membrane composition and stress response systems, which confer resistance to such selective pressures. Similarly, a concentration of 3.5% NaCl inhibits the growth of most Gram-positive and some Gram-negative bacteria. However, *H. pylori* exhibits tolerance to this level of salinity, allowing its selective cultivation under these conditions [36,37,38]. Since each selective growth medium suppresses background bacteria via different mechanisms, using parallel selective formulations improves the probability of recovering fastidious target cells without compromising viability. The development of such food-specific media underscores a critical gap between clinical and food microbiology, as methodologies optimized for gastric specimens are not always directly transferable to complex food matrices. Food and environmental samples often harbor a more diverse and complex microbiota than clinical specimens. While the use of selective media can enhance the efficiency of *H. pylori* isolation, their effectiveness is context-dependent. Culture-based detection of *H. pylori* from food samples remains considerably more challenging than from clinical specimens due to factors such as microbial competition, sample matrix complexity, and the organism’s fastidious growth requirements [2,39,40]. Raw meat contains a diverse and abundant background microflora that can inhibit the growth of *H. pylori* on selective media, further lowering culture sensitivity. Moreover, environmental stresses such as refrigeration, oxygen exposure, and nutrient limitation can induce a viable but non-culturable (VBNC) state, in which cells remain metabolically active but fail to grow on standard media [38]. These combined factors mean that culture methods detect only the recoverable fraction of the population, and the actual prevalence in meat may be higher than observed.

This study compares *H. pylori* prevalence in red meat from different retail sources, including slaughterhouses, butcher shops, and local markets. Although butcher shops showed a higher prevalence (15.5%), but not statistically significant. The differences among sources were not statistically significant (*p* > 0.05), suggesting that contamination risk may be relatively uniform across the retail chain. This pattern reflects in previous study results where source-level differences were insignificant or inconsistent, suggesting that there is a need for developing hygiene standards across all stages. A few studies have directly compared sampling sources, and their results have been mixed. For example, in Isfahan, Iran, Gilani et al. [39] reported an overall prevalence of 5%, with slightly higher rates in slaughterhouse samples (7.3%) than in butcheries (2.7%). Similarly, in Yemen, Almashhadany et al. [23] found a prevalence of 13.8% in raw chicken meat, with no significant difference between slaughterhouse and market sources. Given the limited number of studies worldwide that systematically compare retail sources, the present findings provide valuable data on the distribution of *H. pylori* contamination along the meat supply chain and emphasize the need for consistent sanitary measures at all stages.

This study compared the prevalence of *H. pylori* among different meat types (beef, sheep, and goat). Although beef showed the highest prevalence (15.2%), the differences among meat types were not statistically significant (*p* > 0.05), suggesting that contamination rates may vary rather than following a consistent pattern across species. Reports on *H. pylori* in red meat remain limited; some studies using PCR assays on gastric biopsy specimens reported prevalence of 3% in cattle and 16% in sheep, with no detection in goats [41], while another study using cultural methods reported rates of 11.9% in cows and 6.9% in buffaloes [42]. Additional reports have identified prevalence ranging from 7.8% to 13.8% in poultry meat [23,43], and 12.5% to 32% in minced meat [40,44]. Although PCR confirmed species identity, the absence of strain-level molecular typing limits assessment of genetic diversity among isolates, identification of virulence markers (e.g., *cagA*, *vacA* genotypes) and understanding of antibiotic resistance mechanisms. Therefore, further research is warranted to determine strain-level relatedness, potential zoonotic connections, or the genetic basis of observed antimicrobial resistance patterns [45]. Similarly, DNA-based quantitative methods have been shown to accurately quantify *H. pylori* burden and identify virulence or resistance genes even at low loads. The absence of such quantitative load data limits the interpretation of public health and surveillance implications of contamination [46].

Beyond meat, *H. pylori* has also been detected in a variety of food products and environmental sources, highlighting its wide distribution. The bacterium has been isolated from ready-to-eat foods (13.5%), ready-to-eat fish (15%), chicken and meat sandwiches (8.3% and 20%) and hamburgers (1.4%). It has also been reported in seafood, with prevalence as high as 67% in mussels, 25% in clams, and 8% in cockles [2,15,47]. In dairy products, prevalence (ranging from 4.8 to 20%) has been documented in raw milk from cows, sheep, goats, buffaloes, and camels [48]. Other studies have reported 13.3% prevalence in raw cow milk and 6.7% in retail cow milk [16], with notably high (72.2%) rates in raw cow milk in Japan [49]. Furthermore, *H. pylori* has been detected in vegetables (6–20%), ready-to-eat salads (16.6%), basil (12.5%), radish (7.5%), and lettuce (13.8%) [15,50], as well as in drinking water, seawater, and influent and effluent water samples from drinking water treatment plants, using both culture- and PCR-based methods [15,51,52].

Collectively, these findings, together with the present study, indicate that although prevalence varies across food types, *H. pylori* is consistently detected in multiple food matrices, supporting the hypothesis that foods, including meat, may contribute to potential exposure pathways for humans.

All *H. pylori* isolates in this study were resistant to nalidixic acid and susceptible to cephalothin, a classic phenotypic method used to differentiate *H. pylori* from other Gram-negative organisms, such as *Campylobacter* spp. *H. pylori* typically exhibits resistance to nalidixic acid and susceptibility to cephalothin, whereas *Campylobacter* spp. often display opposite pattern [3]. Findings from the present study align with the previous environmental studies, in which up to 80% of isolates showed similar resistance patterns [53]. However, some poultry-derived strains have demonstrated variability [23]. These differences could be attributed to strain-specific opposite or source-related antimicrobial resistance profiles. Monitoring these phenotypic markers is essential for risk assessment and for informing therapeutic approaches in both clinical and food safety contexts.

The antimicrobial susceptibility testing of 47 *H. pylori* isolates recovered from various meat sources revealed a heterogeneous resistance profile, reflecting the ongoing challenges in managing this pathogen. Amoxicillin (80.8%) and ampicillin (76.6%) showed the highest effectiveness among the tested antibiotics. Their sustained activity may be attributed not only to their pharmacological properties but also to variations in usage patterns across regions, where differences in prescribing practices, regulations, and resistance dynamics could explain the comparatively high effectiveness observed in this study. Moreover, clarithromycin (74.5%) and levofloxacin (78.7%) demonstrated strong efficacy, consistent with regional data. Comparable results have been reported for foodborne *H. pylori* isolates, with relatively high susceptibility rates to clarithromycin (82.3%) and levofloxacin (87.3%) observed in strains recovered from various types of raw milk (bovine, ovine, caprine, buffalo, camel, and donkey) and dairy products (traditional cheese, cream, butter, and ice cream) in Iran [51]. Similarly, *H. pylori* strains isolated from raw poultry meat (chicken, turkey, ostrich) in Shahrekord, Iran, showed susceptibility to clarithromycin (65%) and several fluoroquinolones, including levofloxacin (70%) [54]. However, resistance to clarithromycin is increasing globally due to its widespread use in respiratory and gastrointestinal infections [53]. Similarly, levofloxacin remains effective locally where resistance rates exceeding 30–40% have been reported in parts of Asia and Europe, likely driven by intensive fluoroquinolone use in both clinical and agricultural sectors [54]. Metronidazole resistance (40.4%) emerged as the most prominent concern, consistent with global trends, especially in food and environmental isolates. This resistance is often linked to its extensive use in parasitic and anaerobic bacterial infections, and also *H. pylori*’s ability to inactivate reductase enzymes essential for drug activation [55]. Similarly, moderate resistance levels were also observed for erythromycin (36.2%) and azithromycin (23.4%), suggesting possible cross-resistance within the macrolide class. Comparable resistance profiles have been documented in *H. pylori* strains from poultry and meat products, underscoring the potential impact of antimicrobial use in animal husbandry [52]. The presence of antimicrobial-resistant *H. pylori* isolates in meat products highlights a food safety concern and supports the need for surveillance, although direct zoonotic transmission cannot be inferred from the present data. While antibiotics such as amoxicillin, ampicillin, clarithromycin, and levofloxacin remain effective, the observed resistance to metronidazole and macrolides underscores the importance of prudent antibiotic use and integrated antimicrobial stewardship in both healthcare and food production systems. The presence of antimicrobial-resistant *H. pylori* in food sources poses a significant public health concern. Foodborne transmission of resistant bacterial strains may contribute to the broader dissemination of antimicrobial resistance surveillance within the food production system and support risk-based hygiene interventions. Moreover, resistant strains detected in food animals or food products may represent potential sources of exposure and persistence within the food chain; therefore, monitoring of antimicrobial resistance in foodborne pathogens is warranted to support food safety surveillance and risk assessment [56,57]. Overall, detection of AMR in meat contributes to the foodborne AMR reservoir with potential implications for zoonotic exposure and downstream treatment efficacy, which reinforces the urgent need for antimicrobial stewardship, residue monitoring, and integrated One Health surveillance that includes food matrices.

Although the role of food and water in *H. pylori* transmission remains unclear, clinical and environmental evidence suggests a possible route via ingestion of raw or undercooked foods, including vegetables irrigated with contaminated water [58]. Consuming street food or raw vegetables such as lettuce has been identified as a risk factor for infection [59]. This observation supports the hypothesis that irrigation with fecally contaminated water may facilitate survival of *H. pylori* and contribute to its transmission to humans [60].

Moreover, no statistically significant seasonal variation was observed in this study (*p* > 0.05), the relatively higher prevalence of *H. pylori* in late spring (April–June) may reflect improved survival of the organism under certain environmental conditions. *H. pylori* employs multiple adaptive mechanisms, including stress tolerance to temperature shifts, oxidative stress, and nutrient limitation during slaughtering, processing, and storage [61]. *H. pylori* utilizes flagella for motility and produces urease, which hydrolyzes urea into ammonia, thereby neutralizing gastric acidity and creating a localized microenvironment favorable for colonization. In cattle, sheep, and goats, *H. pylori* primarily colonizes the stomach (abomasum) and may pose a zoonotic risk through the consumption of contaminated milk or direct animal contact. Host adaptation appears to vary among ruminant species, with sheep generally exhibiting higher prevalence, cattle showing moderate colonization, and goats demonstrating lower susceptibility. Infection can result in chronic gastritis and gastric ulceration, similar to disease manifestations in humans. These pathological effects are mediated by key virulence factors, including urease, vacuolating cytotoxin A (*VacA*), and cytotoxin-associated gene A (*CagA*), which collectively induce inflammation, epithelial cell damage, and host immune responses, although disease severity varies among ruminant hosts [62]. Also, the bacterium can enter the VBNC state, remaining metabolically active yet undetectable by culture, which may explain intermittent detection. Additionally, biofilm formation on meat and food-contact surfaces protects against desiccation, disinfectants, and immune responses [63]. These factors support *H. pylori* persistence in raw meat and along the food chain, representing a possible consumer exposure route.

Further studies involving genome-based characterization for strain-level variation and broader epidemiological surveillance are needed to better understand the pathogenic potential, zoonotic linkages and assess the risk of cross-contamination between animal and human isolates and transmission dynamics of *H. pylori* in the foodborne context.

## 5. Conclusions

This study provides the first evidence of *H. pylori* contamination in raw red meat samples from different retail sources in Dhamar, Yemen. Overall, *H. pylori* was recovered from all three selective media at variable rates ranging from 2.4% to 6.6%, with relatively higher recovery on Modified MCA media than on other media. This suggests that MCA can be used for the enhanced recovery and isolation of *H. pylori* from meat. Moreover, *H. pylori* was commonly detected in beef cattle (15.2%) than in goat (12.5%) and sheep (9.6%) meat. Moreover, all isolated strains showed variable susceptibility to antibiotics, suggesting that various environmental factors may impact the susceptibility status of this pathogen. Therefore, regular monitoring of AMR profiles is essential to track emerging resistance trends and develop One Health antimicrobial policies. Also, the results suggest that red meat may act as a matrix supporting the presence of *H. pylori*, which may represent a possible public health concern from a food safety perspective. To mitigate this risk, PCR-based methods have the potential to assess *H. pylori* contamination of meat during the slaughtering process, as they provide complementary information and represent effective tools for monitoring meat contamination and hygienic practices during slaughtering and carcass processing. Similarly, comparative genomics between foodborne and clinical isolates will clarify zoonotic linkages and transmission dynamics. Moreover, proper hygiene practices should be enforced throughout the meat production and supply chain, including at the slaughterhouse and retail levels. Public health education promoting thorough cooking and proper food handling is also essential.

## Figures and Tables

**Table 1:** Number and percentage of *H. pylori* isolates recovered from fresh raw meat samples according to meat type and sampling source.

Meat Type * (Number of Samples)	*H. pylori* Isolates ^§^ *n* (%)	Number of Samples/Positive Number (%) Based on Source of Sample **		
		Slaughterhouses (*n* = 127)	Retail Markets (*n* = 124)	Butcher Shops (*n* = 129)
Beef cattle (*n* = 125)	19 (15.2)	43/6 (13.9)	40/5 (12.5)	42/8 (19.1)
Sheep (*n* = 135)	13 (9.6)	46/4 (8.7)	44/3 (6.8)	45/6 (13.3)
Goat (*n* = 120)	15 (12.5)	38/5 (13.2)	40/4 (10)	42/6 (14.3)
Total (*n* = 380)	47 ^§§^ (12.4)	127/15 (11.8)	124/12 (9.7)	129/20 (15.5)

*n*: Number of samples. *: Differences in *H. pylori* prevalence among meat types were not statistically significant (χ^2^ test, *p* = 0.394). **: Differences among sampling sources were also not statistically significant (χ^2^ test, *p* = 0.362). ^§^: Growth on selective media (MCA = Modified Campy blood agar; BHA = Belo Horizonte agar; EYE = Egg Yolk Emulsion agar). ^§§^: Of 64 putative culture isolates, 47 were confirmed as *H. pylori* using biochemical methods.

**Table 2:** Antimicrobial susceptibility profile of *H. pylori* isolates recovered from various meat types.

Antimicrobial Class	Antimicrobial Agent	Resistance Profile Based on Meat Source			Overall Resistance Profile		
		Beef Cattle ^§^ *n* (%)	Sheep ^§§^ *n* (%)	Goat ^§§§^ *n* (%)	Resistant * n (%)	Intermediate *n* (%)	Susceptible *n* (%)
β-lactams (Penicillin)	Ampicillin (AMP, 10 µg)	4 (21.1)	2 (15.4)	3 (20)	9 (19.1)	2 (4.3)	36 (76.6)
	Amoxicillin (AMX, 10 µg)	4 (21.1)	0 (0.0)	2 (13.3)	6 (12.8)	3 (6.4)	38 (80.8)
Tetracyclines	Tetracycline (TE, 30 µg)	6 (31.6)	3 (23.1)	4 (26.7)	13 (27.7)	5 (10.6)	29 (61.7)
Macrolides	Erythromycin (E, 15 µg)	9 (47.4)	2 (15.4)	6 (40)	17 (36.2)	7 (14.9)	23 (48.9)
	Clarithromycin (CLR, 15 µg)	4 (21.1)	1 (7.7)	2 (13.3)	7 (14.9)	5 (10.6)	35 (74.5)
	Azithromycin (AZM, 30 µg)	5 (26.3)	2 (15.4)	4 (26.7	11 (23.4)	7 (14.9)	29 (61.7)
Nitroimidazoles	Metronidazole (MT, 5 µg)	11 (57.9)	3 (23.1)	5 (33.3)	19 (40.4)	3 (6.4)	25 (53.2)
Fluoroquinolones	Levofloxacin (LE, 5 µg)	3 (15.8)	0 (0.0)	4 (26.7)	7 (14.9)	3 (6.4)	37 (78.7)

*n*: number of isolates. ^§^: Values in parentheses were calculated based on 19/47 isolates from beef cattle meat samples. ^§§^: Values in parentheses were calculated based on 13/47 isolates from Sheep meat samples. ^§§§^: Values in parentheses were calculated based on 15/47 isolates from goat meat samples. *: Values in parentheses were calculated based on the total number of isolates (*n* = 47).

**Table 3:** Temporal distribution of 47 *H. pylori* isolates based on sample source and meat types.

Month ^§^	Total Number of Samples	Number of Positive Samples (%) Based on Sample Source ^§§^				Number of Positive Samples (%) Based on Meat Types ^§§§^			
		Slaughterhouses (*n* = 127)	Retail Markets (*n* = 124)	Butcher Shops (*n* = 129)	Total (*n* = 380)	Beef Cattle (*n* = 125)	Sheep (*n* = 135)	Goat (*n* = 120)	Total (*n* = 380)
January	62	2 (3.2) *	1 (1.6)	2 (3.2)	5 (8.1)	2 (3.2) *	1 (1.6)	2 (3.2)	5 (8.1)
February	63	2 (3.2)	0 (0)	2 (3.2)	4 (6.3)	1 (1.6)	2 (3.2)	1 (1.6)	4 (6.3)
March	63	0 (0)	3 (4.8)	1 (1.6)	4 (6.3)	3 (4.8)	0 (0)	1 (1.6)	4 (6.3)
April	65	4 (6.2)	3 (4.6)	6 (9.2)	13 (20)	5 (7.7)	4 (6.2)	4 (6.2)	13 (20)
May	65	3 (4.6)	3 (4.6)	5 (7.7)	11 (16.9)	4 (6.2)	3 (4.6)	4 (6.2)	11 (16.9)
June	62	4 (6.5)	2 (3.2)	4 (6.5)	10 (16.1)	4 (6.5)	3 (4.8)	3 (4.8)	10 (16.1)
Total	380	15 (11.8)	12 (9.7)	20 (15.5)	47 (12.4)	19 (15.2)	13 (9.6)	15 (12.5)	47 (12.4)

^§^: *p*-value = 0.091 (monthly variation), statistically insignificant; *: The percentages were calculated based on monthly totals; ^§§^: *p*-value = 0.670 (Prevalence by source over time), not statistically significant; ^§§§^: *p*-value = 0.957 (Prevalence by meat type over time), not statistically significant.

## Data Availability

The original contributions presented in this study are included in the article. Further inquiries can be directed to the corresponding authors.
